# Pesticide use and risk of Hodgkin lymphoma: results from the North American Pooled Project (NAPP)

**DOI:** 10.1007/s10552-020-01301-4

**Published:** 2020-04-20

**Authors:** Lidija Latifovic, Laura E. Beane Freeman, John J. Spinelli, Manisha Pahwa, Linda Kachuri, Aaron Blair, Kenneth P. Cantor, Shelia Hoar Zahm, Dennis D. Weisenburger, John R. McLaughlin, James A. Dosman, Punam Pahwa, Stella Koutros, Paul A. Demers, Shelley A. Harris

**Affiliations:** 1 Occupational Cancer Research Centre, Cancer Care Ontario, Toronto, ON Canada; 2 Division of Cancer Epidemiology and Genetics, U.S. National Cancer Institute, Bethesda, MD USA; 3 Population Oncology, BC Centre, Vancouver, BC Canada; 4 School of Population and Public Health, University of British Columbia, Vancouver, BC Canada; 5 Centre for Health Economics and Policy Analysis, McMaster University, Hamilton, ON Canada; 6 Department of Epidemiology & Biostatistics, University of California At San Francisco, San Francisco, CA USA; 7 Shelia Zahm Consulting, Hermon, ME USA; 8 Department of Pathology, City of Hope Medical Center, Duarte, CA USA; 9 University of Saskatchewan, Saskatoon, SK Canada; 10 Public Health Ontario, Toronto, ON Canada; 11 Dalla Lana School of Public Health, University of Toronto, 155 College St, Toronto, ON M5T 3M7 Canada

**Keywords:** Pesticide, Insecticide, Organophosphate, Hodgkin lymphoma, Cancer, Case–control

## Abstract

**Purpose:**

The purpose of this study was to investigate associations between pesticide exposures and risk of Hodgkin lymphoma (HL) using data from the North American Pooled Project (NAPP).

**Methods:**

Three population-based studies conducted in Kansas, Nebraska, and six Canadian provinces (HL = 507, Controls = 3886) were pooled to estimate odds ratios and 95% confidence intervals for single (never/ever) and multiple (0, 1, 2–4, ≥ 5) pesticides used, duration (years) and, for select pesticides, frequency (days/year) using adjusted logistic regression models. An age-stratified analysis (≤ 40/ > 40 years) was conducted when numbers were sufficient.

**Results:**

In an analysis of 26 individual pesticides, ever use of terbufos was significantly associated with HL (OR: 2.53, 95% CI 1.04–6.17). In age-stratified analyses, associations were stronger among those ≤ 40 years of age. No significant associations were noted among those > 40 years old; however, HL cases ≤ 40 were three times more likely to report ever using dimethoate (OR: 3.76 95% CI 1.02–33.84) and almost twice as likely to have ever used malathion (OR: 1.86 95% CI 1.00–3.47). Those ≤ 40 years of age reporting use of 5 + organophosphate insecticides had triple the odds of HL (OR: 3.00 95% CI 1.28–7.03). Longer duration of use of 2,4-D, ≥ 6 vs. 0 years, was associated with elevated odds of HL (OR: 2.59 95% CI 1.34–4.97).

**Conclusion:**

In the NAPP, insecticide use may increase the risk of HL, but results are based on small numbers.

**Electronic supplementary material:**

The online version of this article (10.1007/s10552-020-01301-4) contains supplementary material, which is available to authorized users.

## Introduction

Hodgkin lymphoma (HL) is a cancer of the lymphatic system with an estimated 8500 new cases in the USA and 990 new cases in Canada in 2017 [1, 2]. Men are at slightly greater risk of HL compared to women [3]. Epidemiological evidence suggests a viral etiology with Epstein-Barr virus (EBV), which also causes mononucleosis [3]. Those with a history of EBV infection are at 2–3 times greater risk of developing HL [4]. However, this link is more clearly established for classical Hodgkin lymphoma and less so for the other major subtype, nodular lymphocyte-predominant Hodgkin lymphoma [4]. Family history, genetics (many susceptibility polymorphisms map to genes that affect immune function such as the human leukocyte antigen (HLA) region [5]), autoimmune disorders, immunodeficiency and tobacco use are also associated with HL [3].

The prevalence of lymphatic and hematopoietic cancers is elevated in farmers and agricultural populations [6–10]. Although pesticides are important for the protection of our food supply, it is well documented that human exposure to high doses can lead to serious toxicities [11–14] and there is evidence that exposures at lower levels have adverse effects on health [15, 16]. While many chemicals currently in use have not been reviewed with respect to their carcinogenicity, there are reports that suggest some pesticides might be of concern [17–20]. Suggested associations are with several cancer sites and do not appear to be limited to any specific functional or chemical class [21, 22]. The evidence linking pesticide use to HL is equivocal. A Canadian case–control study (Cross-Canada Study of Pesticides and Health (CCSPH)) reported an increased risk of HL with exposure to multiple insecticides (5 + vs 0 OR: 1.88, 95% CI 0.92–3.87) [23], the insecticide chlorpyrifos (ever vs. never OR: 1.19, 95% CI 1.03–1.37) [24] and the herbicide dichlorprop (ever vs. never OR: 6.35 95% CI 1.56–25.92) [25]. A hospital-based case–control study from France reported increased risks of HL with occupational use of carbamate fungicides (OR: ever vs. never OR: 5.1, 95% CI 1.4–18.4), triazole fungicides (ever vs. never OR: 8.4 95% CI 2.2–32.4), urea derivative herbicides (ever vs. never OR: 10.8 95% CI 2.4–48.1), both organophosphate and organochlorine insecticides (ever organophosphate use vs. never OR: 3.0 95% CI 1.0–9.4; ever organochlorine use vs. never OR: 4.7 95% CI 1.1–20.8), as well as domestic insecticide use (ever vs. never OR: 2.9 95% CI 1.6–5.4) [26]. Domestic insecticide use during pregnancy has been reported to be associated with childhood HL (use during pregnancy vs. no use during pregnancy OR: 4.1 95% CI 1.4–11.8) [27]. Several other studies found no association between HL and pesticide use [28–34].

The North American Pooled Project (NAPP) [35] included case–control studies from a broad geographic range with diverse agricultural practices and different occupational and non-occupational exposures to pesticides. The aim of this analysis was to use these pooled case–control data from the NAPP to investigate possible associations between self-reported pesticide use and HL. The association with the use of multiple pesticides, the association with individual pesticides and the association with duration and frequency of use for select pesticides was investigated.

## Methods

### The North American Pooled Project (NAPP)

The NAPP is composed of four case–control studies conducted in Kansas, Iowa and Minnesota, and Nebraska (1980s) in the United States and the CCSPH (1990s). Data from three of these studies were harmonized to provide larger numbers to evaluate possible associations between agricultural exposures and risk of several lymphatic and hematopoietic cancers. Complete details of study design, participant recruitment, data collection and harmonization are described elsewhere (NAPP [35], Iowa and Minnesota [36], Kansas [37], Nebraska [38] and CCSPH [39]). The questionnaire design in the CCSPH was modeled after the studies in Kansas and Nebraska allowing for efficient harmonization and pooling of data.

### Population selection and outcome ascertainment

The study from Iowa and Minnesota [36] did not recruit HL cases; thus, controls from this study were excluded from this analysis, which includes cases and controls from three of the four NAPP studies. In Kansas, data were obtained from 121 newly diagnosed, pathologist confirmed HL cases (international classification of disease (ICD)-9-code 201), among men aged 21 years and older, identified from a population-based registry covering the state of Kansas from 1976 to 1982 [37]. Population-based controls (*N* = 948) identified via random-digit dialing, Medicare, or state mortality files were frequency matched to cases on age (± 2 years) and vital status. The response rate was 69.9% among HL cases 94.0% among controls. For the Nebraska study, men and women 21 years of age and older, with a first diagnosis of HL from 1983 to 1986, were identified through the Nebraska Lymphoma Study Group and area hospitals in eastern Nebraska [38]. Controls were selected from the same area with 3:1 frequency matching by race, sex, vital status and age (± 2 years) by two-stage random-digit dialing, Medicare, or state mortality files. Response rates were 91.0% for cases and 87.2% for controls. Proxy respondents were used for participants unable to complete study questionnaires independently in Kansas (26.5% cases and 52.3% controls) and Nebraska (22.9% cases and 43.6% controls). In the CCSPH, incident cases among men, 19 years of age and older, with a first diagnosis of HL between 1991 and 1994 were ascertained from cancer registries in six Canadian provinces, except for hospital ascertainment in Quebec [39]. Controls were men selected from provincial health insurance records (Alberta, Saskatchewan, Manitoba and Quebec), telephone listings (Ontario), or voter’s lists (British Columbia) stratified by age (± 2 years) and province. The studies recruited cases with multiple lymphatic and hematopoietic cancers. Response rates were 48.0% for controls and 67.1% for NHL cases. Response rates for HL were not reported. A total of 507 HL cases and 3 886 population-based controls were available from Kansas, Nebraska and the CCSPH. The controls were matched to the overall age groupings of all cancer cases recruited by the NAPP and not Hodgkin lymphoma cases specifically. Therefore, the age distribution varies between the cases and controls included in this analysis.

### Pesticide use in the North American Pooled Project

Pesticide use was self-reported via interviewer-administered questionnaire by telephone in Kansas and Nebraska and by mailed questionnaire in the CCSPH. In the Kansas study, cases and controls, or their proxy respondents, were interviewed by telephone and reported information on pesticides used and the names and locations of companies where pesticides were purchased. To corroborate self-reported pesticide use, pesticide suppliers for 110 participants from the Kansas study were contacted and asked to provided information on the participants’ crops and their herbicide and insecticide purchases. Approximately, 60% of self-reports agreed with suppliers’ records of purchases [37, 40]. In the Nebraska study, blinded telephone interviews conducted by interviewers who were not aware of the participants’ case–control status were used to collect information on pesticide use, years of use, and the average annual number of days of use on the farm [38]. CCSPH questionnaires were modified versions of questionnaires used in the Kansas and Nebraska studies [39]. In a validation pilot study conducted on the modified questionnaires, the suppliers of 27 farmers were contacted for access to their purchase records. Agreement between self-reported pesticide use and pesticide purchase records was reported as excellent [39]. In the CCSPH, pesticide use data were collected using a two-stage approach. Canadian participants who reported 10 or more hours per year of pesticide use on a postal questionnaire and a 15% random sample of the remaining participants were contacted by telephone for a detailed interview to collect information on their use of major classes of pesticides and individual compounds. For each self-reported individual pesticide used, information on ever use (yes/no), duration of use (years), and frequency of use (number of days/year) was collected in Nebraska and CCSPH. Duration and frequency of use of specific pesticide compounds were not collected in Kansas. Participants were prompted to report use of individual pesticides using a list of chemicals and trade names provided in the CCSPH and Nebraska. However, in Kansas this prompt was not employed; instead an open-ended question was used to ask participants to recall the specific chemicals. Use of over 120 different insecticides, herbicides, and fungicides was reported in the NAPP. Information on demographic characteristics, occupation, lifestyle, medical history and other possible cancer risk factors was also collected in the questionnaires.

### Statistical analysis

Descriptive analyses, which included frequencies, means and standard deviation, median and range values, were conducted to determine the distribution of covariates. Wald 95% confidence intervals (CI) and odds ratios (OR) were estimated using logistic regression (Fisher’s scoring method) in SAS v 9.4 (SAS Institute, Cary, NC). Models were selected based on a theoretical consideration of the relationships between variables using the directed acyclic graph approach [41–43] (Supplementary Fig. S1). In addition to the design variables of age, sex, province or state of residence and respondent type, variables considered as potential confounders included level of education, a history of allergies, a history of a doctor diagnosed mononucleosis, family history of lymphatic or hematopoietic cancers and having worked or lived on farmland. A multivariable model was selected using the change-in-estimate approach, with a 10% change in the coefficient estimate considered as meaningful. Final models were adjusted for the design variables of age group (< 30, 30–39, 40–49, 50–59, ≥ 60), sex (male, female), province or state of residence (Nebraska, Kansas, Quebec, Ontario, Manitoba, Saskatchewan, Alberta, British Columbia) and respondent type (proxy, self). Observations with missing adjustment covariate values were removed from the analysis (listwise deletion) for a complete-case analysis (97.5% of data) that included 496 HL cases and 3789 controls.

Exposures to multiple pesticides (0, 1, 2–4, ≥ 5 pesticides), multiple pesticides grouped by functional group (fungicide, herbicide, insecticide) and select major chemical groups (organophosphate, organochlorine, and carbamate insecticides) were considered. Analyses for individual pesticides were performed for compounds with at least five exposed cases. Given the bimodal incidence of HL, suggesting multiple etiologies, effect modification by age, ≤ 40 and > 40 years old [23], was explored for compounds with a sufficient number of exposed cases. Furthermore, when there were enough exposed cases (malathion, methoxychlor, 2,4-dichlorophenoxyacetic acid (2,4-D), glyphosate) the effect of duration of use (years) and frequency of use (days per year) was investigated. In analysis of duration and frequency of pesticide use, observations with missing duration and frequency were dropped from the analysis. P-trend values for duration (0, 3, 9.5 years) and frequency of pesticide use categories (0, 1, 6 days/year) were calculated as Wald statistics from respective logistic regression models.

### Sensitivity analyses

Three sensitivity analyses were performed: (1) re-sampling controls to match the age-frequency distribution of the cases, (2) excluding proxy respondents and (3) fitting models with a random effects parameter for province or state of residence. Controls were resampled to match the age distribution of cases stratified for state or province of residence and age group category. Odds ratios and 95% confidence intervals were estimated using multiple logistic regression adjusted for age category, sex, respondent status and province or state of residence. Results are presented in Supplementary Tables S1–S4. The results from logistic regression models adjusted for age group, sex and province or state of residence excluding proxy respondents are presented in Supplementary Tables S7–S9. Results from mixed logistic regression models, with a random effects parameter for province or state of residence, and adjusted for age, sex and respondent status are presented in Supplementary Tables S10–S13.

### Ethics

The NAPP received ethics approval from the University of Toronto Research Ethics Board and an exemption from the NIH Office of Human Subjects Research Protection. All case–control studies included in the NAPP received ethics approval at the time the studies were conducted, and informed consent was obtained from all individual participants included.

## Results

### Participant characteristics

Cases were on average younger than controls, more likely to have a family history of lymphatic or hematopoietic cancer and more likely to have a history of doctor diagnosed mononucleosis. Educational attainment, having lived or worked on farmland, smoking cigarettes and medically diagnosed allergies were not associated with HL (Table 1). Subtype information was missing for 41.2% of HL cases. However, among those cases with complete subtype information nodular sclerosis was the most common HL subtype in the NAPP overall (26.6%), followed by mixed cellularity (12.4%). Among those 40 and younger with complete HL subtype information, nodular sclerosis (35.6%) was the most common HL subtype. However, among those older than 40 there was a more even distribution of HL subtypes (nodular sclerosis (17.7%), mixed cellularity (14.3%) and other (21.2%)) (Table 2). Subtype information was not collected in the case–control studies from Nebraska and Kansas.

**Table 1:** Characteristics of Hodgkin lymphoma cases and controls in the North American Pooled Project

Characteristic	Cases		Controls				
	*N*	%	*N*	%	OR^a^	95% CI	*p*^b^
Age (years)							
Younger than 30	150	29.6	295	7.6			
30–39	125	24.7	381	9.8			
40–49	80	15.8	415	10.7			
50–59	54	10.7	515	13.3			
60 or older	97	19.1	2250	57.9			
Missing	1	0.2	30	0.8			
Sex							
Male	472	93.1	3179	81.8			
Female	35	6.9	707	18.2			
Original study							
Nebraska	70	13.8	1432	36.9			
Kansas	121	23.9	948	24.4			
CCSPH	316	62.3	1506	38.8			
Respondent type							
Self	422	83.2	2546	65.5			
Proxy	74	14.6	1273	32.8			
Missing	11	2.2	67	1.7			
Education							
Some high school or less	109	21.5	1355	34.9	1.00		
High school	116	22.9	902	23.2	0.97	0.70–1.35	0.97
1–3 years of college	89	17.6	407	10.5	0.90	0.62–1.31	0.67
College graduate or higher	151	29.8	838	21.6	1.02	0.74–1.42	0.86
Missing	42	8.3	384	9.9			
Ever lived or worked on farmland							
No	272	53.7	1530	39.4	1.00		
Yes	235	46.4	2356	60.6	0.89	0.73–1.10	0.29
Ever smoked cigarettes							
No	137	27.0	656	16.9	1.00		
Yes	308	60.8	2317	59.6	1.14	0.89–1.47	0.30
Missing	62	12.2	913	23.5			
History of lymphatic/hematopoietic cancer in immediate family							
No	457	90.1	3621	93.2	1.00		
Yes	26	5.2	151	3.9	**1.95**	**1.21**–**3.15**	**0.0064**
Missing	24	4.7	114	2.9			
Ever diagnosed with allergies							
No	359	70.8	3023	77.8	1.00		
Yes	121	23.9	756	19.5	1.09	0.85–1.39	0.48
Missing	27	5.3	107	2.8			
Ever diagnosed with mononucleosis							
No	433	85.4	3681	94.7			
Yes	42	8.3	83	2.1	**1.99**	**1.31**–**3.01**	**0.0010**
Missing	32	6.3	122	3.1			
Total	507	100.0	3886	100.0			

*OR* odds ratio; *CI* confidence interval

^a^Adjusted for age group, sex, province/state of residence and respondent type

^b^*p* value from Wald chi-square test with 1 degree of freedom

^c^Bold text indicates statistically significant odds ratios

**Table 2:** Hodgkin lymphoma subtypes in the North American Pooled Project

HL subtype ^a^	Total (*N* = 507)		≤ 40 years old (*N* = 275)		> 40 years old (*N* = 231) ^b^	
	*N*	*%*	*N*	*%*	*N*	*%*
Nodular sclerosis	135	26.6	98	35.6	41	17.7
Mixed cellularity	63	12.4	30	10.9	33	14.3
Other	100	19.7	46	16.7	49	21.2
Missing ^c^	209	41.2	101	36.7	108	46.8

^a^ICD morphology code: 9663, 9664, 9665, 9667 = nodular sclerosis; 9652 = mixed cellularity

^b^One case with missing age

^c^Subtype information was not collected by the studies from Nebraska and Kansas

### Use of multiple pesticides

The use of multiple pesticides as a group was not associated with HL, nor was the use of multiple herbicides, fungicides, or organochlorine insecticides (Table 3). Hodgkin’s lymphoma cases had 1.85 (95% CI 1.05–3.24) times the odds of reporting use of five or more insecticides than controls and 1.59 (95% CI 0.96–2.63) times the odds of reporting use of two or more organophosphate insecticides than controls. Cases were also more likely to report use of two or more carbamate insecticides (OR: 2.56, 95% CI 1.07, 6.15). Among those older than 40, no statistically significant associations were observed with the use of multiple pesticides. However, among those 40 and younger, the use of five or more insecticides (OR: 2.45, 95% CI 0.93–6.44) and two or more organophosphate insecticides (OR: 2.96, 95% CI 1.33–6.61, *p*-trend < 0.01) was significantly associated with HL. Furthermore, the interaction with age and ever use of organophosphate insecticides was statistically significant (*p* = 0.01).

**Table 3:** Adjusted odds ratios and corresponding 95% confidence intervals for Hodgkin lymphoma from a stratified analysis for age, ≤ 40 and > 40 years old, and number of self-reported pesticides used, by type of pesticide and chemical class, in the North American Pooled Project

	Overall					≤ 40 years old					> 40 years old					
	Cases	Controls				Cases	Controls				Cases	Controls				
	496	3789				269	660				227	3129				
	N exposed		OR	95% CI		N exposed		OR	95% CI		N exposed		OR	95% CI		*p*-int
Pesticides																0.47
0	340	2513	1.00			439	178	1.00			162	2074	1.00			
1	40	436	0.85	0.59	1.24	70	24	1.01	0.61	1.68	16	366	0.73	0.43	1.25	
2–4	77	542	1.05	0.79	1.40	105	44	1.04	0.69	1.57	33	437	1.03	0.70	1.54	
5 +	39	298	0.98	0.67	1.44	46	23	1.17	0.67	2.06	16	252	0.81	0.47	1.41	
*p*-trend				0.99				0.62					0.56			
Fungicides																
0	480	3719	1.00													
1	11	59	0.823	0.41	1.65											
2 +	5	11	1.81	0.585	5.60											
*p*-trend			0.71													
Herbicides																0.24
0	370	2907	1.00			195	484	1.00			175	2423	1.00			
1	68	512	1.01	0.75	1.36	36	108	0.88	0.57	1.34	32	404	1.11	0.75	1.66	
2–4	40	254	0.97	0.66	1.42	25	47	1.15	0.67	1.97	15	207	0.85	0.48	1.49	
5 +	18	116	1.25	0.72	2.19	13	21	1.53	0.71	3.28	5	95	0.90	0.35	2.29	
*p*-trend			0.69					0.43					0.75			
Insecticides																0.5
0	357	2657	1.00			190	475	1.00			167	2182	1.00			
1	70	662	0.96	0.71	1.28	42	118	1.00	0.67	1.51	28	544	0.90	0.59	1.38	
2–4	51	355	1.00	0.71	1.40	28	56	1.13	0.68	1.86	23	299	0.89	0.56	1.41	
5 +	18	115	1.85	1.05	3.24	9	11	2.45	0.93	6.44	9	104	1.61	0.78	3.35	
*p*-trend			0.30					0.21					0.84			
Organophosphate insecticides																0.01
0	443	3474	1.00			235	614	1.00			208	2860	1.00			
1	31	179	1.03	0.68	1.58	19	31	1.29	0.70	2.38	12	148	0.87	0.47	1.61	
2 +	22	136	1.59	0.96	2.63	15	15	2.96	1.33	6.61	7	121	0.86	0.39	1.89	
*p*-trend			0.12					< 0.01					0.58			
Organochlorine insecticides																0.31
0	427	3315	1.00			223	591	1.00			194	2724	1.00			
1	42	268	0.98	0.68	1.42	22	50	0.92	0.53	1.59	20	218	1.05	0.64	1.73	
2 +	27	206	1.09	0.70	1.71	14	19	1.53	0.73	3.20	13	187	0.92	0.51	1.68	
p-trend			0.78					0.46					0.89			
Carbamate insecticides																
0	471	3635	1.00													
1	17	124	1.01	0.59	1.75											
2 +	8	30	2.56	1.07	6.15											
*p*-trend			0.13													

*OR* odds ratio; *CI* confidence interval; bold text indicates a statistically significant result at alpha = 0.05

^a^Adjusted for age group, sex, province/state of residence and respondent status

^b^Where at least 5 exposed cases in each age category

^c^Joint test for product term including age dichotomized at 40 and pesticide category

## Ever use of individual pesticides

Self-reported ever use of 16 individual insecticides was investigated and results are presented in Table 4. In the NAPP overall, ever use of terbufos was statistically significantly associated with HL (OR: 2.58, 95% CI 1.06–6.25). Among those 40 and younger, elevated odds ratios were noted for dimethoate and malathion, although the estimate for dimethoate was based on small numbers (8 exposed cases, 5 exposed controls). Furthermore, the interactions between age and malathion (*p*-interaction = 0.005), age and dimethoate (*p* = 0.03) and age and chlordane (*p* = 0.03) were statistically significant. Among those older than 40, no statistically significant associations were found.

**Table 4:** Adjusted odds ratios and corresponding 95% confidence intervals for Hodgkin lymphoma and ever use of specific self-reported insecticides and a stratified analysis for age, ≤ 40 and > 40 years old, in the North American Pooled Project

	Overall					≤ 40 years old ^c^					> 40 years old ^c^					
	N exposed					N exposed					N exposed					
	Cases	Controls	OR^b^	95% CI		Cases	Controls	OR^b^	95% CI		Cases	Controls	OR^b^	95% CI		p-int^d^
Pesticide^a^																
Organophosphate																
Chlorpyrifos	6	21	1.83	0.69	4.89											
Diazinon	14	89	1.58	0.84	2.94	8	9	2.08	0.76	5.64	6	80	1.23	0.52	2.94	0.22
Dimethoate	13	70	1.39	0.73	2.65	8	5	3.43	1.04	11.34	5	65	0.83	0.32	2.12	0.03
Famphur	6	33	2.47	0.96	6.38											
Fonofos	6	44	2.07	0.81	5.28											
Malathion	36	198	1.21	0.81	1.81	26	29	1.91	1.07	3.43	10	169	0.68	0.35	1.33	0.005
Phorate	7	51	1.78	0.75	4.24											
Terbufos	7	42	2.58	1.06	6.25											
Organochlorine																
Aldrin	6	62	1.77	0.72	4.33											
Chlordane	19	158	0.80	0.47	1.34	13	18	1.40	0.66	3.00	6	140	0.47	0.20	1.10	0.03
Dieldrin	5	51	1.77	0.66	4.70											
DDT	20	217	1.14	0.69	1.88	6	8	2.03	0.67	6.13	14	209	1.05	0.59	1.86	0.19
Lindane	11	80	1.70	0.85	3.42	4	5	1.96	0.50	7.68	7	75	1.86	0.81	4.22	0.70
Methoxychlor	43	208	0.94	0.64	1.36	26	56	0.86	0.51	1.45	17	152	0.98	0.57	1.69	0.81
Carbamate																
Carbaryl	16	78	1.74	0.96	3.16	11	13	2.46	1.04	5.81	5	65	1.06	0.42	2.71	0.14
Carbofuran	14	93	1.29	0.70	2.40											

*OR* odds ratio; *CI* confidence interval; *int* interaction; bold text indicates statistically significant result at alpha = 0.05

^a^If ≥ 5 exposed cases then included in analysis

^b^Adjusted for age group, sex, province/state of residence, respondent status

^c^Where at least 5 exposed cases in each age category

^d^Joint test for product term including age dichotomized at 40 and the specific insecticide modeled

The association with HL was investigated for ever use of 12 individual herbicides (Table 5). No elevated odds ratios or statistically significant associations were observed in the NAPP overall. In an age-stratified analysis, HL cases 40 and younger were two times more likely to report ever use of dicamba (OR: 2.09, 95% CI 0.91–4.81) and 1.69 times more likely to report ever use of trifluralin (95% CI 0.78–3.67). In general, elevated odds were not observed for the individual herbicides investigated among those older than 40 years.

**Table 5:** Adjusted odds ratios and corresponding 95% confidence intervals for Hodgkin lymphoma and ever use of specific self-reported herbicides and a stratified analysis for age, ≤ 40 and > 40 years old, in the North American Pooled Project

	Overall					≤ 40 years old ^c^					> 40 years old^c^					
	N exposed					N exposed					N exposed					
	Cases	Controls	OR^b^	95%CI		Cases	Controls	OR^b^	95%CI		Cases	Controls	OR^b^	95%CI		p-int^d^
Pesticide^a^																
Phenoxy herbicide																
2,4-D	83	536	1.08	0.82	1.43	50	90	1.29	0.86	1.92	33	446	0.88	0.59	1.31	0.09
2,4,5-T	6	69	0.82	0.34	1.97											
MCPA	12	62	0.98	0.50	1.93	7	10	1.20	0.43	3.38	5	52	0.90	0.34	2.37	0.36
Carbamate herbicide																
Diallate	7	29	0.94	0.38	2.35											
Triallate	6	16	1.85	0.65	5.26											
Other herbicide																
Alachlor	11	100	1.25	0.63	2.48											
Atrazine	14	141	1.02	0.56	1.85	7	29	0.77	0.32	1.84	7	112	1.31	0.58	2.95	0.57
Bromoxynil	11	48	0.93	0.46	1.91											
Dicamba	16	86	1.28	0.71	2.30	12	14	2.09	0.91	4.81	4	72	0.71	0.25	1.99	0.05
Glyphosate	40	184	1.00	0.68	1.49	28	51	1.03	0.61	1.73	12	133	0.98	0.52	1.84	0.44
Propachlor	7	61	1.27	0.55	2.96											
Trifluralin	17	90	1.31	0.73	2.35	14	18	1.69	0.78	3.67	3	72	0.77	0.24	2.54	0.08
Fungicide																
Captan	6	20	1.32	0.50	3.52											
Thiram	9	39	0.88	0.40	1.94											

*OR* odds ratio; *CI* confidence interval; *int* interaction; bold text indicates statistically significant result at alpha = 0.05

^a^If ≥ 5 exposed cases then included in analysis

^b^Adjusted for age group, sex, province/state of residence, respondent status

^c^Where at least 5 exposed cases in each age category

^d^Joint test for product term including age dichotomized at 40 and the specific insecticide modeled

HL was not associated with ever use of captan and thiram, the fungicides with enough exposed cases to be considered in the individual analysis (Table 5).

## Duration of use and frequency of use of select pesticides

Results for duration of use and frequency of use of malathion, methoxychlor, 2,4-D, and glyphosate in relation to the risk of HL are presented in Table 6. Numbers of exposed cases were small, and CIs were wide. No obvious exposure–response trends were observed in the analysis for duration and frequency of use of any specific pesticide. However, among the 40 and younger age group duration of use of 1–5 years and frequency of use of 1–2 days/year of malathion was statistically significantly associated with HL (*p*-interaction with age = 0.03). Duration of use of 2,4-D in the NAPP overall tended to be slightly elevated, but no statistically significant associations were observed. We noted a statistically significant interaction with age for 2,4-D (p-interaction < 0.001). In those 40 and younger, HL cases had 2.58 times the odds of reporting ≥ 6 years of 2,4-D use than controls (95% CI 1.38–4.83, *p*-trend < 0.01).

**Table 6:** Adjusted odds ratios and corresponding 95% confidence intervals for Hodgkin lymphoma and duration (years used) and frequency of use (days/year) of select pesticides in the North American Pooled Project

	Overall					≤ 40 years old					> 40 years old					
	N exposed					N exposed					N exposed					
Pesticide	Cases	Controls	OR	95% CI		Cases	Controls	OR	95% CI		Cases	Controls	OR	95% CI		*p*-int
Organophosphate insecticide																
Malathion																
Duration of use																0.03
0	460	3591	1.00			631	243	1.00			217	2960	1.00			
1–5	22	77	1.71	1.00	2.92	12	16	2.80	1.25	6.25	6	65	0.96	0.40	2.30	
≥ 6 years	10	73	0.92	0.46	1.86	10	7	1.47	0.53	4.09	3	63	0.49	0.15	1.59	
Missing	4	48														
*p*-trend			0.63					0.20					0.32			
Frequency of use																0.03
0	460	3596	1.00			243	633				217	2963	1.00			
1–2	19	79	1.28	0.73	2.25	14	10	2.46	1.03	5.85	5	69	0.64	0.25	1.64	
≥ 3 days / year	7	51	0.94	0.41	2.17	5	8	1.48	0.46	4.73	2	43	0.49	0.12	2.05	
Missing	10	63														
*p*-trend			0.81					0.58					0.87			
Organochlorine insecticide																
Methoxychlor																
Duration of use																0.82
0	453	3581	1.00			243	604	1.00			210	2977	1.00			
1–5	10	55	0.87	0.43	1.78	6	15	0.97	0.37	2.59	4	40	0.92	0.32	2.64	
≥ 6 years	25	125	0.90	0.56	1.45	14	35	0.75	0.39	1.47	11	90	1.00	0.51	1.94	
Missing	8	28														
*p*-trend			0.9													
Frequency of use																0.66
0	453	3581	1.00					1.00			210	2977	1.00			
1–2	8	72	0.52	0.24	1.12			0.33	0.11	0.99	4	50	0.69	0.24	1.94	
≥ 3 days / year	27	101	1.20	0.75	1.92			1.17	0.61	2.25	11	72	1.26	0.64	2.48	
Missing	8	35														
*p*-trend			0.41					0.54					0.46			
Phenoxy herbicide																
2,4-D																
Duration of use																< 0.001
0	413	3253	1.00			219	570	1.00			194	2683	1.00			
1–5	27	140	0.91	0.57	1.46	18	44	0.85	0.47	1.56	9	96	0.94	0.46	1.95	
≥ 6 years	38	265	1.18	0.80	1.74	25	23	2.58	1.38	4.83	13	242	0.63	0.35	1.14	
Missing	18	131														
*p*-trend			0.31					< 0.01					0.22			
Frequency of use																0.08
0	413	3253	1.00			219	570				194	2683				
1–2	29	186	0.81	0.52	1.26	20	40	0.98	0.54	1.78	9	146	0.56	0.28	1.14	
≥ 3 days / year	29	163	1.16	0.75	1.82	18	24	1.69	0.87	3.30	11	139	0.81	0.42	1.56	
Missing	25	187														
*p*-trend			0.43					0.09					0.55			
Organophosphate herbicide																
Glyphosate																
Duration of use																0.21
0	456	3605	1.00			241	609	1.00			215	2996	1.00			
1–5	23	102	0.93	0.56	1.53	16	36	0.79	0.42	1.50	7	66	1.06	0.47	2.39	
≥ 6 years	15	57	1.38	0.73	2.58	11	9	2.47	0.97	6.24	4	48	0.94	0.33	2.69	
Missing	2	25														
*p*-trend			0.43					0.29					0.91			
Frequency of use																0.80
0	456	3606	1.00			241	610	1.00			215	2996	1.00			
1–2	25	103	1.02	0.62	1.66	17	29	1.01	0.52	1.94	8	74	1.03	0.48	2.23	
≥ 3 days / year	14	42	1.53	0.79	2.96	10	13	1.68	0.70	4.02	4	29	1.40	0.48	4.12	
Missing	1	38														
*p*-trend			0.2					0.29					0.53			

*OR* odds ratio; *CI* confidence interval

^a^If ≥ 5 exposed cases then included in analysis

^b^Adjusted for age group, sex, province/state of residence, respondent status

^c^Where at least 5 exposed cases in each age category

^d^Joint test for product term including age dichotomized at 40 and the specific insecticide modeled; Bold text indicates a statistically significant result at alpha = 0.05

## Sensitivity analyses

In general, the results of the sensitivity analyses are qualitatively like those from the primary analysis with statistically significant associations noted for use of five or more insecticides and the use of 2 or more carbamate insecticides. From logistic regression models, on data where controls were age-frequency re-matched to the HL cases, the OR for use of 5 + insecticides (relative to 0) is 1.75, 95% CI 0.92–3.31 and the OR for use of 2 + carbamate insecticides (relative to 0) is 3.37, 95% CI 1.16–9.77 (Supplementary Table S1). Similarly, the odds ratio for those reporting use of 5 + insecticides relative to those who reported not having used any insecticides, from mixed logistic regression models that included a random effects parameter for province or state of residence, is 1.76, 95% CI 1.01–3.07. From the same Supplementary Table S10, the OR for those who reported having used 2 + carbamate insecticides relative to never users is 2.40, 95% CI 1.01–5.73. Odds ratios from the analysis that excluded proxy respondents were 1.93 for the use of 5 + insecticides (95% CI 1.05–3.55) and 2.61 for the use of 2 + carbamate insecticides (95% CI 1.00–6.86) (Supplementary Table S7). Additionally, while not statistically significant, elevated odds ratios were noted for all the individual organophosphate and carbamate insecticides investigated, the organochlorine insecticides aldrin, dieldrin, lindane, the herbicides triallate and trifluralin and the fungicide captan.

## Discussion

The epidemiologic evaluation of cancer risk resulting from pesticide exposure is challenging because of intermittent exposures of varying levels and changes in use patterns over time. We attempted to address these complexities by assessing associations using a variety of analytical approaches: by functional group (herbicide, fungicide, insecticide), by major chemical class (organophosphate, organochlorine, and carbamate), and when data were available, by the use of different exposure metrics (ever vs never, duration of use and frequency of use) for individual compounds within these classes.

The analyses of broad groupings of pesticides showed a few interesting trends. First, ORs for those 40 years of age or younger tended to be elevated in comparison to those in the over 40 age group. No analysis for the over 40 group showed a statistically significant trend with the number of different pesticides used, in contrast to the 40 and under group. Second, a simple counting of pesticides used in the different groups was sometimes associated with an increased risk. This points to the need to perform more sophisticated analyses in additional studies, with available information on the timing of pesticide use, to address the issue of multiple and overlapping exposures.

Our results showed an elevated risk of Hodgkin lymphoma with exposure to multiple organophosphate insecticides among those under 40 years of age. Navaranjan et al. [23] had previously noted this in the CCSPH for acetylcholinesterase inhibitors and Orsi et al., from a French case–control study [26]. The two main classes of cholinesterase inhibiting pesticides are organophosphates and carbamates. We observed an elevated odds ratio for the organophosphate terbufos, and for those 40 and younger for dimethoate and malathion. Using our pooled NAPP data, we did not observe much of the previously reported association between HL and the organophosphorous insecticide chlorpyrifos [24], although there were only five exposed cases. HL risk in relation to dichlorprop [25] was not investigated in this study as information on the use of this herbicide was only collected by the CCSPH. Similarly, we were not able to confirm the previous associations with the chemical groups’ pyrethrin insecticides and picoline, amide and urea herbicides [26] as we did not have exposure information for these pesticides.

Pesticides can affect cell proliferation [44], cause cytotoxicity [45–49], act as endocrine disruptors [50] and alter the immune response [51–53] to produce tumor-promoting effects [54–57]. Dimethoate and carbaryl were grouped as possible carcinogens with limited evidence in animals and malathion and 2,4-D as genotoxic toxicants by Gunier et al. [58] based on information from the California legislature mandated Pesticide Use Report [58]. Terbufos and fonofos have been linked to lung cancer, leukemia, non-Hodgkin’s lymphoma and prostate cancer [59–63]. Use of terbufos was also associated with a non-significant increase in breast cancer risk in a prospective study evaluating the use of organophosphates and cancer at multiple sites among women [64]. Malathion has been linked with thyroid cancer [64], inconsistently with non-Hodgkin lymphoma [64–66] and prostate cancer [66]. Carbaryl was reported to be associated with melanoma, non-Hodgkin lymphoma [21, 67] and prostate cancer [68] and 2,4-D inconsistently with non-Hodgkin lymphoma, respiratory cancers [69] and prostate cancer [68, 70, 71].

The effect modification by age observed in this study may be due to the distribution of different subtypes of HL by age. Nodular sclerosis typically develops in teens and young adults 15–35 years of age. In our study population nodular sclerosis was 2.4 (95% CI 1.21–4.57) times more common among younger HL cases (≤ 40 years of age). It is possible that the differences in the association between HL and pesticide use observed between the two age groups represent different etiologies in HL subtype. However, because many our study participants were missing subtype information, we did not have enough exposed cases to make any conclusive comments about the association between pesticide use and subtype of HL. A history of doctor diagnosed mononucleosis was also more common among those 40 and younger than those older than 40 (≤ 40: 9.5%, > 40: 1.1%, *p* value < 0.0001). Additionally, there were some differences in pesticide exposure patterns by age. A higher proportion of those 40 and younger reported having used fungicides (3.7% vs. 1.7%, *p* = 0.02), the herbicides diallate, bromoxynil, glyphosate, and trifluralin and the insecticide methoxychlor (Supplementary Table S5). In contrast, a higher proportion of those older than 40 reported use of organochlorine insecticides, specifically aldrin, dieldrin, DDT, and lindane.

Although we found some elevations in risk associated with pesticide use, limitations of this study must be acknowledged and include exposure measurement error and potentially uncontrolled confounding. The studies pooled in the NAPP relied on self-reported information on the personal use of pesticides, often years prior to the interview and in some instances information on use was reported by proxy respondents. Non-differential exposure misclassification would undoubtedly occur and has been demonstrated in methodological investigations of the NAPP and other farm populations [72–75]. Non-differential misclassification would tend to bias estimates of relative risk toward the null [75]. However, the effects of measurement error, particularly in combination with unmeasured confounding can be unpredictable. Because the NAPP is composed of case–control studies, there is also a possibility of differential exposure misclassification, which can bias relative risks toward or away from the null depending upon the location and magnitude of the error. A specific methodological effort in the Nebraska case–control study [40] found no evidence for case-response bias regarding pesticides in that study. Previously, year-to-year repeatability based on questionnaires for 11 commonly used pesticides in a group of 4 088 pesticide applicators was reported to be high for self-reported pesticide use, varying from 70 to 90%. Agreement for duration and frequency was lower, varying from 50 to 59% for days of use per year and 50–77% for duration of use in years. However, inter-individual variation in exposure in a single day of pesticide use can be high. It has previously been reported that the number of acres treated in a day by farmers, the number of pesticides mixed, the pounds of active pesticide ingredients handled, and daily urinary concentrations of pesticide metabolites can vary considerably between individuals [76–78]. Nevertheless, previous studies suggest that while self-reported pesticide use information lacks the precision required to detect dose–response effects, ever users and frequent users of individual pesticides can be differentiated from never users or infrequent users with reasonable accuracy. Approximately 31% of pesticide use reports in the NAPP were from proxy respondents. A previous study investigating the accuracy of information collected from proxy respondents showed that proxies were more likely to report use of fewer pesticides, unknown use or no use of specific pesticides while famers were three to five times more likely to report use of five or more pesticides [40]. We performed a sensitivity analysis excluding proxy respondents. Qualitatively, results were like those reported for the primary analysis. For Kansas and the CCSPH, response rates for cases were lower than those for controls. If the choice to participate in the study was related to pesticide use (i.e., cases who enrolled in the study were higher users of pesticides) then this is a form of selection bias that would impact the estimated magnitude of the association between pesticide use and HL. If cases were more likely to participate because they had used pesticides, this would overestimate the exposed proportion among cases and overestimate the true effect.

Confounding is always a concern in epidemiologic studies. These pooled studies, however, collected information on many potential risk factors and statistical adjustment was employed in the analysis, although there are not many risk factors that are likely to be associated with pesticide use to cause confounding [79]. Given the age distribution of the cases and controls there is the potential for residual confounding by age in our study population. The controls were generally older than the cases with a relative lack of younger controls to match the case distribution. We performed a sensitivity analysis, presented in Supplementary Tables S1-S4, in which we resampled the controls to match the age distribution of the cases stratified by state or province of residence. Qualitatively, the results were the same in the resampled subset as the full data. We elected to use the full data to maximize statistical power. Furthermore, many comparisons were made, some based on small numbers, and these may generate chance findings. Although the pooling of data for the NAPP resulted in an increased overall sample size, the numbers of exposed participants for many pesticides was still low, limiting our ability to assess their effect on HL risk. Few participants reported long-term exposure to the insecticides investigated. While selection bias, residual bias due to unmeasured confounding, and measurement error likely have an impact on the reported results, a formal quantitative bias analysis is not warranted given the imprecision that is already reflected in the confidence intervals presented for the observed association estimates.

It is important to note that the pesticide exposure information used in this study was collected in the late 1980s to early 1990s. However, many of the pesticides investigated are still in use today including: captan, carbaryl, 2,4-D, glyphosate, diazinon, dicamba, dimethoate, chlorpyrifos, malathion, atrazine, thiram, trifluralin, and alachlor. Finally, given that < 7% of participants were female, the results of this study cannot be generalized to women who make up a considerable proportion of HL cases in adults.

Strengths of this study include the large sample size of the NAPP, which did allow us to investigate a larger number of individual pesticides as compared to previous studies, as well as to investigate different metrics of pesticide exposure, and to consider age differences that are associated with HL subtypes. The availability of medical history, including family history of cancer and lymphatic or hematopoietic cancer, prior diagnosis with allergy (food and drug), asthma, hay fever, mononucleosis, rheumatoid arthritis and tuberculosis allowed us to explore and adjust for possible confounding. Furthermore, the exposure ascertainment methodology employed in the CCHS included the mailing of a list of pesticides with both trade and generic chemical names to participants followed by a telephone interview, permitting the collection of more comprehensive pesticide exposure information [39].

Synergistic interactions are a concern as pesticides are currently tested and regulated on a single compound basis. The presence of synergistic effects when multiple pesticides are used such that they jointly exert a larger effect than predicted is an important consideration for risk assessment given that pesticides exposures co-occur in agricultural or other settings. A review of 73 reported pesticide synergistic interactions, 69 binary and the remaining consisting of combinations of three to eight compounds, from 36 experimental studies, showed that organophosphate and carbamate insecticides (cholinesterase inhibitors), azole fungicides, triazine herbicides and pyrethroid insecticides were overrepresented in the synergistic mixtures [80]. In future studies, if combinations of pesticides that are likely to induce synergistic interaction, or even additivity, can be identified, this information can be used to inform pesticide use practices, mitigate exposure and improve risk assessment.

Our results suggest possible associations between HL and insecticide exposures. Assessment of risk by several approaches, including general functional categories, exposure to specific chemical classes followed by assessment of risk for specific chemical compounds demonstrates the complexity of the relationships between pesticide exposures and HL risk. Although individual pesticides could be related to HL, evaluation of specific combinations of exposure may also be warranted.

## Electronic supplementary material

Below is the link to the electronic supplementary material.

Supplementary file1 (DOCX 562 kb)
