# Supplementary material for: Pesticide use and risk of Hodgkin lymphoma: results from the North American Pooled Project (NAPP)
Source: Cancer Causes Control. 2020 Apr 20;31(6):583–99. doi: 10.1007/s10552-020-01301-4 (PMC7183499; doi:10.1007/s10552-020-01301-4)
Supplement: Supplementary file 1 — Supplementary file1 (DOCX 562 kb) [file 10552_2020_1301_MOESM1_ESM.docx]

**SUPPLEMENTARY MATERIALS**

**ARTICLE TITLE**

Pesticide use and risk of Hodgkin lymphoma: results from the North American Pooled Project (NAPP)

**JOURNAL NAME**

Cancer Causes & Control

**AUTHORS**

Lidija Latifovic^1,11^, Laura E. Beane Freeman^2^, John J. Spinelli^3,4^, Manisha Pahwa^1,5^, Linda Kachuri^1,6^, Aaron Blair^2^, Kenneth P. Cantor^2^, Shelia Hoar Zahm^7^, Dennis D. Weisenburger^8^, John R. McLaughlin^1,10,11^, James A. Dosman^9^, Punam Pahwa^9^, Stella Koutros^2^, Paul A. Demers^1,11^, and Shelley A. Harris^1,11^

**AFFILIATIONS**

^1^ Occupational Cancer Research Centre, Cancer Care Ontario, Toronto, ON, Canada

^2^ Division of Cancer Epidemiology and Genetics, U.S. National Cancer Institute, Bethesda, MD, USA

^3^ Population Oncology, BC Centre, Vancouver, BC, Canada

^4^ School of Population and Public Health, University of British Columbia, Vancouver, BC, Canada

^5^ Centre for Health Economics and Policy Analysis, McMaster University, Hamilton, Canada

^6^ Department of Epidemiology & Biostatistics, University of California at San Francisco, San Francisco, CA, USA

^7^ Shelia Zahm Consulting, Hermon, ME, USA

^8^ Department of Pathology, City of Hope Medical Center, Duarte, CA, USA

^9^ University of Saskatchewan, Saskatoon, SK, Canada

^10^ Public Health Ontario, Toronto, ON, Canada

^11^ Dalla Lana School of Public Health, University of Toronto, Toronto, ON, Canada

**CORRESPONDING AUTHOR**

Shelley Harris

Dalla Lana School of Public Health

University of Toronto

155 College St

Toronto, ON, M5T 3M7

E-mail: [shelley.harris@utoronto.ca](mailto:shelley.harris@utoronto.ca)

**SUPPLEMENTARY MATERIALS**

**SUPPLEMENTARY FIGURES**

**Figure S1.** Directed acyclic graph for the hypothesized association between variables in the relationship between pesticide use and Hodgkin lymphoma.


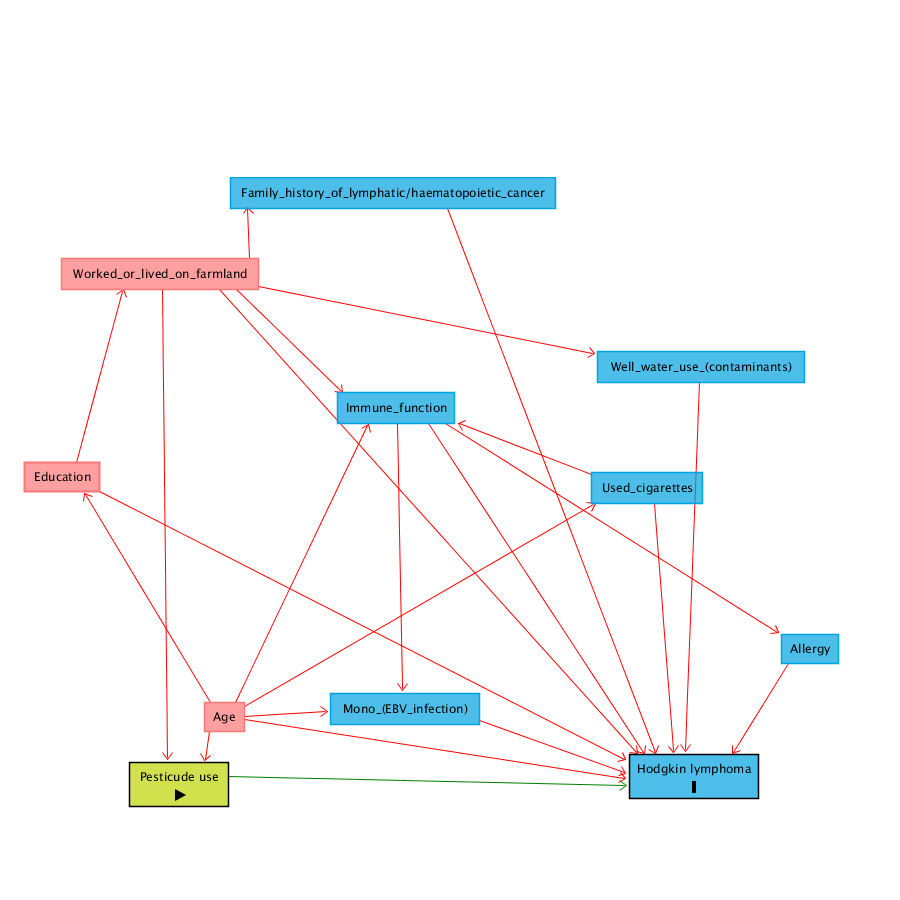


**Notes:** Minimal sufficient adjustment set for estimating the total effect of pesticide use on Hodgkin lymphoma: age and worked or lived on farmland. This diagram was generated using DAGitty.net ^1^

**Figure S2.** Correlation plot with Spearman correlation coefficients for individual pesticide exposures in the North American Pooled Project.


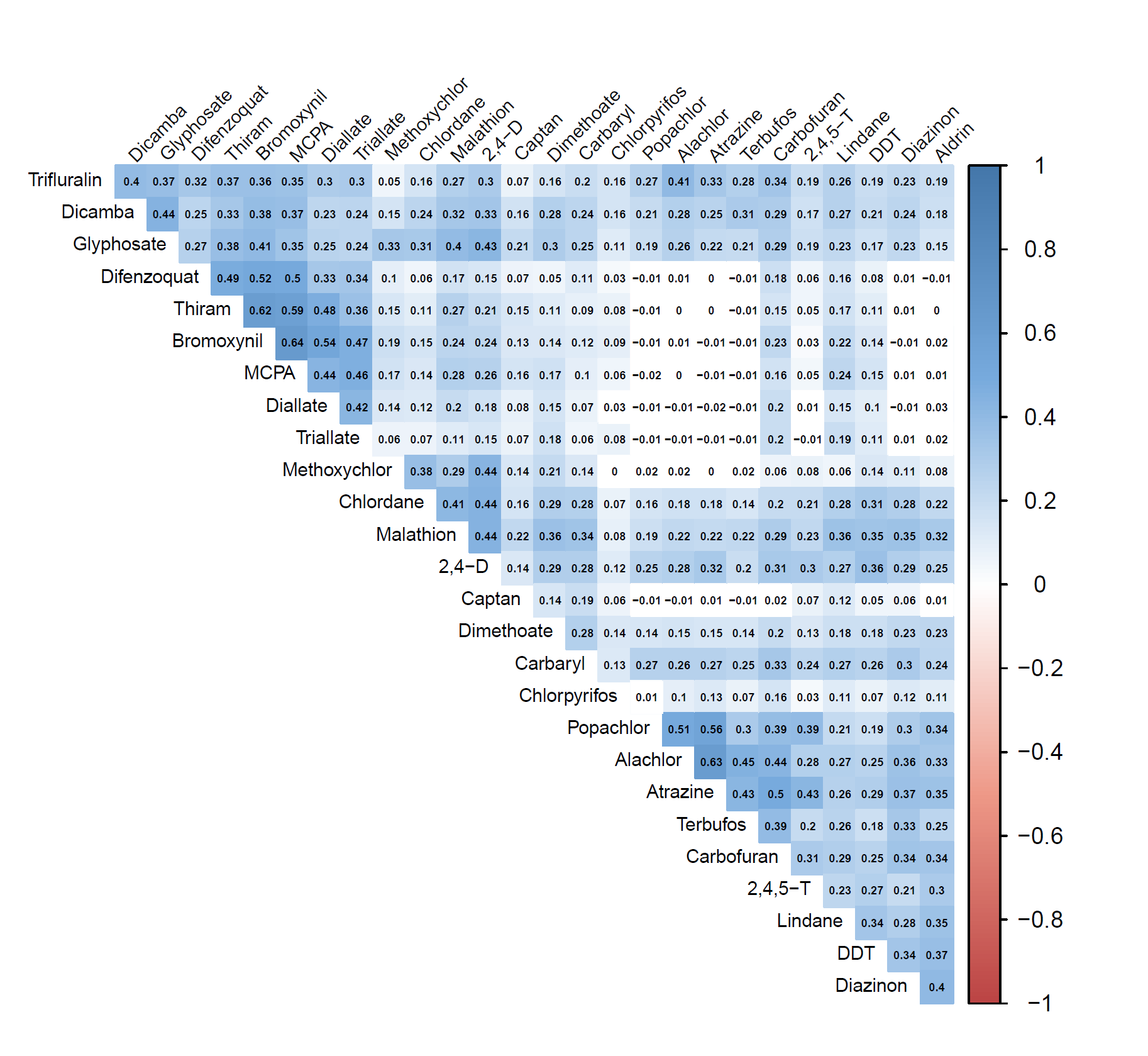


**Figure S3.** Missing data pattern for design [age, proxy respondent and location(province or state of residence)] and medical history variables (history of doctor diagnosed mononucleosis and family history of lymphatic or haematopoietic cancer) in the North American Pooled Project.

**
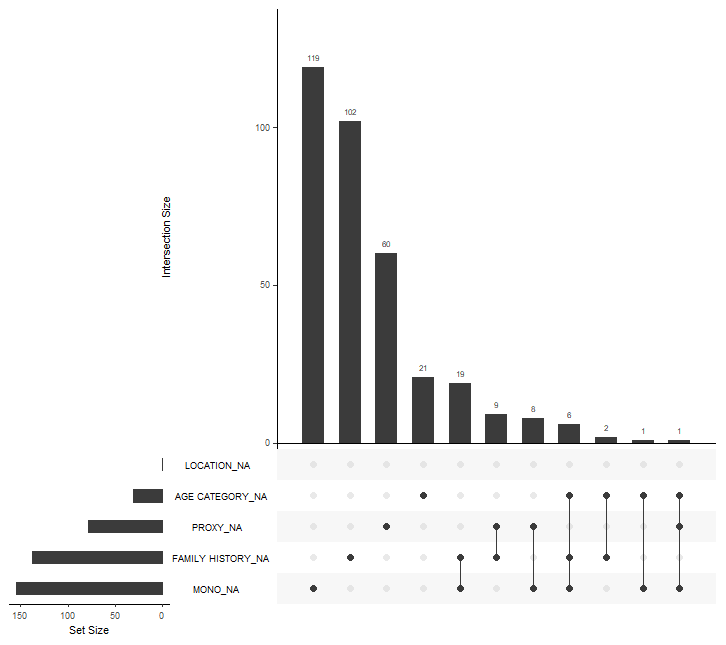
**

**SUPPLEMENTARY TABLES**

**Table S1.** Odds ratios and 95% confidence intervals for the association between number of pesticides used by functional and chemical group and Hodgkin lymphoma in the full data and for the subset of the data with controls that were age-frequency re-matched to the Hodgkin lymphoma cases in the North American Pooled Project.

|  |  |  |  |  | **Full Data** | |  |  |  | **Age-frequency**  **re-matched controls** | | |
| --- | --- | --- | --- | --- | --- | --- | --- | --- | --- | --- | --- | --- |
|  | **Cases** | | **Controls** | | **OR** ^a^ | **95%CI** |  | **Controls** | | **OR ^a^** | **95%CI** | |
|  | *N* | *%* | *N* | *%* |  |  |  | *N* | *%* |  |  |  |
| **Pesticides** |  |  |  |  |  |  |  |  |  |  |  |  |
| 0 | 348 | 68.6 | 2594 | 66.8 | 1.00 |  |  | 695 | 65.5 | 1.00 |  |  |
| 1 | 40 | 7.9 | 439 | 11.3 | 0.85 | 0.59 | 1.23 | 108 | 10.2 | 0.86 | 0.58 | 1.28 |
| 2-4 | 79 | 15.6 | 550 | 14.2 | 1.07 | 0.81 | 1.42 | 171 | 16.1 | 0.99 | 0.73 | 1.34 |
| 5+ | 40 | 7.9 | 303 | 7.8 | 1.00 | 0.68 | 1.45 | 87 | 8.2 | 0.92 | 0.60 | 1.40 |
| **Fungicides** |  |  |  |  |  |  |  |  |  |  |  |  |
| 0 | 490 | 96.7 | 3812 | 98.1 | 1.00 |  |  | 1033 | 97.4 | 1.00 |  |  |
| 1 | 12 | 2.4 | 62 | 1.6 | 0.88 | 0.45 | 1.71 | 24 | 2.3 | 0.74 | 0.36 | 1.55 |
| 2+ | 5 | 1.0 | 12 | 0.3 | 1.76 | 0.58 | 5.41 | 4 | 0.4 | 1.96 | 0.51 | 7.50 |
| **Herbicides** |  |  |  |  |  |  |  |  |  |  |  |  |
| 0 | 378 | 74.6 | 2991 | 77.0 | 1.00 |  |  | 783 | 73.8 | 1.00 |  |  |
| 1 | 70 | 13.8 | 518 | 13.3 | 1.03 | 0.77 | 1.38 | 160 | 15.1 | 0.96 | 0.70 | 1.32 |
| 2-4 | 41 | 8.1 | 259 | 6.7 | 0.98 | 0.67 | 1.43 | 81 | 7.6 | 0.95 | 0.63 | 1.43 |
| 5+ | 18 | 3.6 | 118 | 3.0 | 1.24 | 0.71 | 2.16 | 37 | 3.5 | 1.09 | 0.59 | 2.01 |
| **Insecticides** |  |  |  |  |  |  |  |  |  |  |  |  |
| 0 | 365 | 72.0 | 2739 | 70.5 | 1.00 |  |  | 740 | 69.8 | 1.00 |  |  |
| 1 | 72 | 14.2 | 671 | 17.3 | 0.97 | 0.73 | 1.30 | 184 | 17.3 | 0.94 | 0.69 | 1.28 |
| 2-4 | 51 | 10.1 | 359 | 9.2 | 1.00 | 0.71 | 1.40 | 110 | 10.4 | 0.92 | 0.64 | 1.33 |
| 5+ | 19 | 3.8 | 117 | 3.0 | **1.89** | **1.09** | **3.27** | 27 | 2.5 | 1.75 | 0.92 | 3.31 |
| **Organophosphate ins.** |  |  |  |  |  |  |  |  |  |  |  |  |
| 0 | 453 | 89.4 | 3566 | 91.8 | 1.00 |  |  | 965 | 91.0 | 1.00 |  |  |
| 1 | 31 | 6.1 | 181 | 4.7 | 1.02 | 0.67 | 1.55 | 65 | 6.1 | 0.90 | 0.56 | 1.42 |
| 2+ | 23 | 4.5 | 139 | 3.6 | 1.62 | 0.99 | 2.65 | 31 | 2.9 | **2.02** | **1.12** | **3.63** |
| **Organochlorine ins.** |  |  |  |  |  |  |  |  |  |  |  |  |
| 0 | 435 | 85.8 | 3401 | 87.5 | 1.00 |  |  | 918 | 86.5 | 1.00 |  |  |
| 1 | 44 | 8.7 | 276 | 7.1 | 1.01 | 0.70 | 1.44 | 87 | 8.2 | 0.98 | 0.66 | 1.45 |
| 2+ | 28 | 5.5 | 209 | 5.4 | 1.13 | 0.73 | 1.76 | 56 | 5.3 | 0.96 | 0.59 | 1.56 |
| **Carbamate ins.** |  |  |  |  |  |  |  |  |  |  |  |  |
| 0 | 481 | 94.9 | 3729 | 96.0 | 1.00 |  |  | 1010 | 95.2 | 1.00 |  |  |
| 1 | 18 | 3.6 | 126 | 3.2 | 1.05 | 0.62 | 1.80 | 44 | 4.2 | 0.86 | 0.49 | 1.53 |
| 2+ | 8 | 1.6 | 31 | 0.8 | **2.45** | **1.03** | **5.84** | 7 | 0.7 | **3.37** | **1.16** | **9.77** |

**Notes:** ^a^ adjusted for age group, sex, province or state of residence, respondent status; ins., insecticides; bold numbers indicate a statistically significant result at alpha=0.05

**Table S2.** Odds ratios and 95% confidence intervals for selected insecticides in the full data and for the subset of the data with controls that were age-frequency re-matched to the Hodgkin lymphoma cases in the North American Pooled Project.

|  |  | |  | | **Full Data** | | |  | | **Age-frequency**  **re-matched controls** | | |
| --- | --- | --- | --- | --- | --- | --- | --- | --- | --- | --- | --- | --- |
| **Insecticide ^a^** | **Cases** | | **Controls** | | **OR ^b^** | **95%CI** | | **Controls** | | **OR ^b^** | **95%CI** |  |
|  | *N* | *%* | *N* | *%* |  |  |  | *N* | *%* |  |  |  |
| **Terbufos** |  |  |  |  |  |  |  |  |  |  |  |  |
| Never | 500 | 98.6 | 3844 | 98.9 |  |  |  | 1050 | 99.0 |  |  |  |
| Ever | 7 | 1.4 | 42 | 1.1 | **2.58** | **1.06** | **6.26** | 11 | 1.0 | 2.53 | 0.93 | 6.88 |
| **Phorate** |  |  |  |  |  |  |  |  |  |  |  |  |
| Never | 500 | 98.6 | 3835 | 98.7 |  |  |  | 1043 | 98.3 |  |  |  |
| Ever | 7 | 1.4 | 51 | 1.3 | 1.78 | 0.74 | 4.23 | 18 | 1.7 | 1.49 | 0.59 | 3.73 |
| **Methoxychlor** |  |  |  |  |  |  |  |  |  |  |  |  |
| Never | 462 | 91.1 | 3671 | 94.5 |  |  |  | 976 | 92.0 |  |  |  |
| Ever | 45 | 8.9 | 215 | 5.5 | 0.96 | 0.67 | 1.39 | 85 | 8.0 | 0.89 | 0.60 | 1.33 |
| **Malathion** |  |  |  |  |  |  |  |  |  |  |  |  |
| Never | 470 | 92.7 | 3685 | 94.8 |  |  |  | 996 | 93.9 |  |  |  |
| Ever | 37 | 7.3 | 201 | 5.2 | 1.22 | 0.82 | 1.82 | 65 | 6.1 | 1.11 | 0.71 | 1.73 |
| **Lindane** |  |  |  |  |  |  |  |  |  |  |  |  |
| Never | 496 | 97.8 | 3805 | 97.9 |  |  |  | 1040 | 98.0 |  |  |  |
| Ever | 11 | 2.2 | 81 | 2.1 | 1.68 | 0.84 | 3.37 | 21 | 2.0 | 1.13 | 0.52 | 2.45 |
| **Fonofos** |  |  |  |  |  |  |  |  |  |  |  |  |
| Never | 501 | 98.8 | 3842 | 98.9 |  |  |  | 1051 | 99.9 |  |  |  |
| Ever | 6 | 1.2 | 44 | 1.1 | 2.07 | 0.81 | 5.27 | 1 | 0.1 | 2.43 | 0.84 | 7.00 |
| **Flyspray** |  |  |  |  |  |  |  |  |  |  |  |  |
| Never | 493 | 97.2 | 3688 | 94.9 |  |  |  | 1007 | 94.9 |  |  |  |
| Ever | 14 | 2.8 | 198 | 5.1 | 0.68 | 0.38 | 1.23 | 54 | 5.1 | 0.63 | 0.34 | 1.18 |
| **Famphur** |  |  |  |  |  |  |  |  |  |  |  |  |
| Never | 501 | 98.8 | 3853 | 99.2 |  |  |  | 1050 | 99.0 |  |  |  |
| Ever | 6 | 1.2 | 33 | 0.9 | 2.47 | 0.96 | 6.37 | 11 | 1.0 | 2.03 | 0.73 | 5.70 |
| **Dimethoate** |  |  |  |  |  |  |  |  |  |  |  |  |
| Never | 493 | 97.2 | 3813 | 98.1 |  |  |  | 1045 | 98.5 |  |  |  |
| Ever | 14 | 2.8 | 73 | 1.9 | 1.43 | 0.77 | 2.68 | 16 | 1.5 | 1.74 | 0.82 | 3.68 |
| **Dieldrin** |  |  |  |  |  |  |  |  |  |  |  |  |
| Never | 502 | 99.0 | 3835 | 98.7 |  |  |  | 1053 | 99.3 |  |  |  |
| Ever | 5 | 1.0 | 51 | 1.3 | 1.78 | 0.67 | 4.73 | 8 | 0.7 | 1.47 | 0.46 | 4.69 |
| **Diazinon** |  |  |  |  |  |  |  |  |  |  |  |  |
| Never | 493 | 97.2 | 3796 | 97.7 |  |  |  | 1042 | 98.2 |  |  |  |
| Ever | 14 | 2.8 | 90 | 2.3 | 1.57 | 0.84 | 2.93 | 19 | 1.8 | 1.84 | 0.89 | 3.81 |
| **DDT** |  |  |  |  |  |  |  |  |  |  |  |  |
| Never | 486 | 95.9 | 3667 | 94.4 |  |  |  | 1012 | 95.4 |  |  |  |
| Ever | 21 | 4.1 | 219 | 5.6 | 1.19 | 0.73 | 1.93 | 49 | 4.6 | 0.99 | 0.57 | 1.71 |
| **Chlorpyrifos** |  |  |  |  |  |  |  |  |  |  |  |  |
| Never | 501 | 98.8 | 3865 | 99.5 |  |  |  | 1052 | 99.2 |  |  |  |
| Ever | 6 | 1.2 | 21 | 0.5 | 1.81 | 0.68 | 4.83 | 9 | 0.8 | 1.81 | 0.61 | 5.35 |
| **Chlordane** |  |  |  |  |  |  |  |  |  |  |  |  |
| Never | 487 | 96.1 | 3723 | 95.8 |  |  |  | 1014 | 95.6 |  |  |  |
| Ever | 20 | 3.9 | 163 | 4.2 | 0.82 | 0.50 | 1.37 | 47 | 4.4 | 0.75 | 0.43 | 1.31 |
| **Carbofuran** |  |  |  |  |  |  |  |  |  |  |  |  |
| Never | 493 | 97.2 | 3793 | 97.6 |  |  |  | 1031 | 97.2 |  |  |  |
| Ever | 14 | 2.8 | 93 | 2.4 | 1.29 | 0.70 | 2.40 | 30 | 2.8 | 1.17 | 0.59 | 2.29 |
| **Carbaryl** |  |  |  |  |  |  |  |  |  |  |  |  |
| Never | 490 | 96.7 | 3806 | 97.9 |  |  |  | 1040 | 98.0 |  |  |  |
| Ever | 17 | 3.4 | 80 | 2.1 | **1.80** | **1.01** | **3.21** | 21 | 2.0 | 1.95 | 1.00 | 3.82 |
| **Aldrin** |  |  |  |  |  |  |  |  |  |  |  |  |
| Never | 500 | 98.6 | 3824 | 98.4 |  |  |  | 1050 | 99.0 |  |  |  |
| Ever | 7 | 1.4 | 62 | 1.6 | 1.98 | 0.85 | 4.62 | 11 | 1.0 | 1.73 | 0.64 | 4.66 |

**Notes:** ^a^ selected if ≥ 5 cases; ^b^ adjusted for age group, sex, province or state of residence, respondent status; bold numbers indicate a statistically significant result at alpha=0.05

**Table S3.** Odds ratios and 95% confidence intervals for selected herbicides in the full data and the subset of data with controls that were age-frequency re-matched to the Hodgkin lymphoma cases in the North American Pooled Project.

|  |  | |  | | **Full Data** | | |  | | **Age-frequency**  **re-matched controls** | | |
| --- | --- | --- | --- | --- | --- | --- | --- | --- | --- | --- | --- | --- |
| **Herbicide ^a^** | **Cases** | | **Controls** | | **OR ^b^** | **95%CI** | | **Controls** | | **OR ^b^** | **95%CI** |  |
|  | *N* | *%* | *N* | *%* |  |  |  | *N* | *%* |  |  |  |
| **Alachlor** |  |  |  |  |  |  |  |  |  |  |  |  |
| Never | 496 | 97.8 | 3786 | 97.4 |  |  |  | 1032 | 97.3 |  |  |  |
| Ever | 11 | 2.2 | 100 | 2.6 | 1.25 | 0.63 | 2.48 | 29 | 2.7 | 1.39 | 0.66 | 2.93 |
| **Atrazine** |  |  |  |  |  |  |  |  |  |  |  |  |
| Never | 493 | 97.2 | 3745 | 96.4 |  |  |  | 1018 | 96.0 |  |  |  |
| Ever | 14 | 2.8 | 141 | 3.6 | 1.02 | 0.56 | 1.86 | 43 | 4.1 | 0.98 | 0.52 | 1.87 |
| **Bromoxynil** |  |  |  |  |  |  |  |  |  |  |  |  |
| Never | 496 | 97.8 | 3838 | 98.8 |  |  |  | 1039 | 97.9 |  |  |  |
| Ever | 11 | 2.2 | 48 | 1.2 | 0.94 | 0.46 | 1.92 | 22 | 2.1 | 0.73 | 0.34 | 1.60 |
| **2,4-D** |  |  |  |  |  |  |  |  |  |  |  |  |
| Never | 421 | 83.0 | 3339 | 85.9 |  |  |  | 899 | 84.7 |  |  |  |
| Ever | 86 | 17.0 | 547 | 14.1 | 1.10 | 0.84 | 1.45 | 162 | 15.3 | 1.07 | 0.79 | 1.44 |
| **Diallate** |  |  |  |  |  |  |  |  |  |  |  |  |
| Never | 500 | 98.6 | 3857 | 99.3 |  |  |  | 1050 | 99.0 |  |  |  |
| Ever | 7 | 1.4 | 29 | 0.8 | 0.94 | 0.38 | 2.34 | 11 | 1.0 | 0.78 | 0.29 | 2.14 |
| **Dicamba** |  |  |  |  |  |  |  |  |  |  |  |  |
| Never | 491 | 96.8 | 3797 | 97.7 |  |  |  | 1035 | 97.6 |  |  |  |
| Ever | 16 | 3.2 | 89 | 2.3 | 1.24 | 0.69 | 2.23 | 26 | 2.5 | 1.21 | 0.62 | 2.37 |
| **Difenzoquat** |  |  |  |  |  |  |  |  |  |  |  |  |
| Never | 501 | 98.8 | 3867 | 99.5 |  |  |  | 1050 | 99.0 |  |  |  |
| Ever | 6 | 1.2 | 19 | 0.5 | 1.39 | 0.52 | 3.75 | 11 | 1.0 | 0.78 | 0.28 | 2.21 |
| **Glyphosate** |  |  |  |  |  |  |  |  |  |  |  |  |
| Never | 466 | 91.9 | 3697 | 95.1 |  |  |  | 983 | 92.7 |  |  |  |
| Ever | 41 | 8.1 | 189 | 4.9 | 1.01 | 0.69 | 1.49 | 78 | 7.4 | 0.90 | 0.59 | 1.37 |
| **MCPA** |  |  |  |  |  |  |  |  |  |  |  |  |
| Never | 495 | 97.6 | 3823 | 98.4 |  |  |  | 1036 | 97.6 |  |  |  |
| Ever | 12 | 2.4 | 63 | 1.6 | 0.97 | 0.49 | 1.90 | 25 | 2.4 | 0.69 | 0.33 | 1.45 |
| **Popachlor** |  |  |  |  |  |  |  |  |  |  |  |  |
| Never | 500 | 98.6 | 3825 | 98.4 |  |  |  | 1047 | 98.7 |  |  |  |
| Ever | 7 | 1.4 | 61 | 1.6 | 1.28 | 0.55 | 2.96 | 14 | 1.3 | 1.76 | 0.68 | 4.54 |
| **2,4,5-T** |  |  |  |  |  |  |  |  |  |  |  |  |
| Never | 501 | 98.8 | 3815 | 98.2 |  |  |  | 1038 | 97.8 |  |  |  |
| Ever | 6 | 1.2 | 71 | 1.8 | 0.81 | 0.34 | 1.94 | 23 | 2.2 | 0.59 | 0.23 | 1.48 |
| **Triallate** |  |  |  |  |  |  |  |  |  |  |  |  |
| Never | 501 | 98.8 | 3870 | 99.6 |  |  |  | 1054 | 99.3 |  |  |  |
| Ever | 6 | 1.2 | 16 | 0.4 | 1.85 | 0.65 | 5.24 | 7 | 0.7 | 1.14 | 0.36 | 3.57 |
| **Trifluralin** |  |  |  |  |  |  |  |  |  |  |  |  |
| Never | 490 | 96.7 | 3795 | 97.7 |  |  |  | 1029 | 97.0 |  |  |  |
| Ever | 17 | 3.4 | 91 | 2.3 | 1.30 | 0.72 | 2.35 | 32 | 3.0 | 1.14 | 0.60 | 2.16 |

**Notes:** ^a^ selected if ≥ 5 cases; ^b^ adjusted for age group, sex, province or state of residence, respondent status

**Table S4.** Odds ratios and 95% confidence intervals for selected fungicides in the full data and for the subset of the data with controls that were age-frequency re-matched to the Hodgkin lymphoma cases in the North American Pooled Project.

|  |  | |  | | **Full Data** | | |  | | **Age-frequency**  **re-matched controls** | | |
| --- | --- | --- | --- | --- | --- | --- | --- | --- | --- | --- | --- | --- |
| **Fungicide ^a^** | **Cases** | | **Controls** | | **OR ^b^** | **95%CI** | | **Controls** | | **OR ^b^** | **95%CI** |  |
|  | *N* | *%* | *N* | *%* |  |  |  | *N* | *%* |  |  |  |
| **Captan** |  |  |  |  |  |  |  |  |  |  |  |  |
| Never | 500 | 98.6 | 3862 | 99.4 |  |  |  | 1056 | 99.5 |  |  |  |
| Ever | 7 | 1.4 | 24 | 0.6 | 1.40 | 0.56 | 3.48 | 5 | 0.5 | 2.44 | 0.76 | 7.88 |
| **Thiram** |  |  |  |  |  |  |  |  |  |  |  |  |
| Never | 498 | 98.2 | 3847 | 99.0 |  |  |  | 1041 | 98.1 |  |  |  |
| Ever | 9 | 1.8 | 39 | 1.0 | 0.88 | 0.40 | 1.94 | 20 | 1.9 | 0.62 | 0.27 | 1.44 |

**Notes:** ^a^ selected if ≥ 5 cases; ^b^ adjusted for age group, sex, province or state of residence, respondent status

**Table S5.** Odds ratios (OR) and 95% confidence intervals (CI) for associations between pesticide use and Hodgkin lymphoma sub-types of mixed cellularity (MC) and nodular sclerosis (NS) relative to other sub-types in the North American Pooled Project.

| **Variable** | | **Sub-type** | **OR** | **95% CI** | |
| --- | --- | --- | --- | --- | --- |
| **Age** | |  |  |  |  |
| **<40 vs** | **≥40** |  |  |  |  |
| 26 | 29 | Other | 1.00 |  |  |
| 29 | 26 | MC | 1.17 | 0.54 | 2.54 |
| 94 | 41 | NS | **2.35** | **1.21** | **4.57** |
| **Mono** |  |  |  |  |  |
| **Yes** | **vs. No** |  |  |  |  |
| 3339 | 831 | Other | 1.00 |  |  |
| 39 | 90 | MC | **4.11** | **1.73** | **9.76** |
| 33 | 30 | NS | **4.23** | **2.26** | **7.91** |
| **Use of multiple pesticides** | | |  |  |  |
| **1 vs** | **0** |  |  |  |  |
| 2 | 42 | Other | 1.00 |  |  |
| 5 | 40 | MC | 2.39 | 0.42 | 13.52 |
| 7 | 94 | NS | 1.39 | 0.27 | 7.17 |
| **2-4 vs** | **0** |  |  |  |  |
| 10 | 42 | Other | 1.00 |  |  |
| 7 | 40 | MC | 0.75 | 0.25 | 2.23 |
| 19 | 94 | NS | 0.80 | 0.33 | 1.94 |
| **5+ vs** | **0** |  |  |  |  |
| 1 | 42 | Other | 1.00 |  |  |
| 3 | 40 | MC | 2.85 | 0.25 | 32.88 |
| 15 | 94 | NS | 6.13 | 0.69 | 54.55 |
| **Use of multiple fungicides** | | |  |  |  |
| **1 vs** | **0** |  |  |  |  |
| 2 | 53 | Other | 1.00 |  |  |
| 2 | 53 | MC | 0.90 | 0.11 | 7.14 |
| 10 | 125 | NS | 1.63 | 0.32 | 8.27 |
| **Use of multiple herbicides** | | |  |  |  |
| **1 vs** | **0** |  |  |  |  |
| 9 | 43 | Other | 1.00 |  |  |
| 6 | 43 | MC | 0.67 | 0.21 | 2.12 |
| 15 | 98 | NS | 0.82 | 0.32 | 2.09 |
| **2+ vs** | **0** |  |  |  |  |
| 3 | 43 | Other | 1.00 |  |  |
| 6 | 43 | MC | 1.86 | 0.40 | 8.88 |
| 22 | 98 | NS | 2.34 | 0.61 | 8.99 |
| **Use of multiple insecticides** | | |  |  |  |
| **1 vs** | **0** |  |  |  |  |
| 5 | 43 | Other | 1.00 |  |  |
| 6 | 44 | MC | 0.54 | 0.15 | 1.93 |
| 13 | 98 | NS | 0.60 | 0.21 | 1.73 |
| **2+ vs** | **0** |  |  |  |  |
| 5 | 43 | Other | 1.00 |  |  |
| 6 | 44 | MC | 1.32 | 0.35 | 5.01 |
| 24 | 98 | NS | 2.33 | 0.77 | 7.06 |
| **Use of multiple OP insecticides** | | |  |  |  |
| **1 vs** | **0** |  |  |  |  |
| 4 | 51 | Other | 1.00 |  |  |
| 6 | 49 | MC | 1.70 | 0.42 | 6.89 |
| 21 | 114 | NS | 2.08 | 0.63 | 6.81 |
| **Use of multiple OC insecticides** | | |  |  |  |
| **1 vs** | **0** |  |  |  |  |
| 10 | 45 | Other | 1.00 |  |  |
| 5 | 50 | MC | 0.41 | 0.12 | 1.37 |
| 28 | 107 | NS | 1.20 | 0.51 | 2.85 |
| **Ever use of individual pesticides** | | | | | |
| **Methoxychlor** | |  |  |  |  |
| **Ever vs** | **Never** |  |  |  |  |
| 10 | 45 | Other | 1.00 |  |  |
| 4 | 51 | MC | 0.38 | 0.11 | 1.33 |
| 21 | 114 | NS | 0.83 | 0.35 | 2.02 |
| **DDT** | |  |  |  |  |
| **Ever vs** | **Never** |  |  |  |  |
| 1 | 54 | Other | 1.00 |  |  |
| 1 | 54 | MC | 0.96 | 0.05 | 17.66 |
| 6 | 129 | NS | 2.69 | 0.28 | 25.95 |
| **Glyphosate** | |  |  |  |  |
| **Ever vs** | **Never** |  |  |  |  |
| 3 | 52 | Other | 1.00 |  |  |
| 6 | 49 | MC | 2.29 | 0.49 | 10.65 |
| 21 | 114 | NS | 2.77 | 0.73 | 10.62 |
| **Dicamba** | |  |  |  |  |
| **Ever vs** | **Never** |  |  |  |  |
| 1 | 54 | Other | 1.00 |  |  |
| 1 | 54 | MC | 0.73 | 0.04 | 15.13 |
| 6 | 129 | NS | 1.90 | 0.18 | 19.76 |
| **2,4-D** | |  |  |  |  |
| **Ever vs** | **Never** |  |  |  |  |
| 11 | 44 | Other | 1.00 |  |  |
| 6 | 49 | MC | 0.43 | 0.14 | 1.35 |
| 25 | 110 | NS | 0.70 | 0.30 | 1.68 |

**Notes:** Other: ICD morphology coded 9663, 9664 and 9665; Bold numbers indicate a statistically significant result at alpha=0.05

**Table S6.** Distribution of pesticide exposures across age dichotomized at 40 years old in the North American Pooled Project.

|  | **Age group** | | | |  |
| --- | --- | --- | --- | --- | --- |
|  | **≤ 40 years old** | | **> 40 years old** | |  |
|  | N | % | N | % | p-value ^a^ |
| **Multiple pesticides** |  |  |  |  | 0.33 |
| 0 | 617 | 66.4 | 2236 | 66.6 |  |
| 1 | 94 | 10.1 | 382 | 11.4 |  |
| 2-4 | 149 | 16.0 | 470 | 14.0 |  |
| 5+ | 69 | 7.4 | 268 | 8.0 |  |
| **Multiple fungicides** |  |  |  |  | **0.02** |
| 0 | 901 | 97.0 | 3298 | 98.3 |  |
| 1 | 21 | 2.3 | 49 | 1.5 |  |
| 2+ | 7 | 0.8 | 9 | 0.3 |  |
| **Multiple herbicides** |  |  |  |  | 0.06 |
| 0 | 679 | 73.1 | 2598 | 77.4 |  |
| 1 | 144 | 15.5 | 436 | 13.0 |  |
| 2-4 | 72 | 7.8 | 222 | 6.6 |  |
| 5+ | 34 | 3.7 | 100 | 3.0 |  |
| **Multiple insecticides** |  |  |  |  | 0.27 |
| 0 | 665 | 71.6 | 2349 | 70.0 |  |
| 1 | 160 | 17.2 | 572 | 17.0 |  |
| 2-4 | 84 | 9.0 | 322 | 9.6 |  |
| 5+ | 20 | 2.2 | 113 | 3.4 |  |
| **Multiple OP insecticides** |  |  |  |  | 0.54 |
| 0 | 849 | 91.4 | 3068 | 91.4 |  |
| 1 | 50 | 5.4 | 160 | 4.8 |  |
| 2+ | 30 | 3.2 | 128 | 3.8 |  |
| **Multiple OC insecticides** |  |  |  |  | **0.02** |
| 0 | 824 | 88.7 | 2918 | 87.0 |  |
| 1 | 72 | 7.8 | 238 | 7.1 |  |
| 2+ | 33 | 3.6 | 200 | 6.0 |  |
| **Multiple carbamate insecticides** |  |  |  |  |  |
| 0 | 883 | 95.1 | 3223 | 96.0 | 0.25 |
| 1 | 34 | 3.7 | 107 | 3.2 |  |
| 2+ | 12 | 1.3 | 26 | 0.8 |  |
| **Individual fungicides** |  |  |  |  |  |
| Thiram | 17 | 1.8 | 31 | 0.9 | **0.02** |
| Captan | 10 | 1.1 | 16 | 0.5 | **0.04** |
| **Individual phenoxy herbicides** |  |  |  |  |  |
| 2,4-D | 140 | 15.1 | 479 | 14.3 | 0.54 |
| 2,4,5-T | 15 | 1.6 | 60 | 1.8 | 0.72 |
| MCPA | 17 | 1.8 | 57 | 1.7 | 0.79 |
| **Individual carbamate herbicides** |  |  |  |  |  |
| Diallate | 13 | 1.4 | 23 | 0.7 | **0.04** |
| Triallate | 5 | 0.5 | 17 | 0.5 | 0.90 |
| **Other individual herbicides** |  |  |  |  |  |
| Alachlor | 26 | 2.8 | 85 | 2.5 | 0.65 |
| Atrazine | 36 | 3.9 | 119 | 3.6 | 0.63 |
| Bromoxynil | 22 | 2.4 | 37 | 1.1 | **0.03** |
| Dicamba | 26 | 2.8 | 76 | 2.3 | 0.34 |
| Glyphosate | 79 | 8.5 | 145 | 4.3 | **<0.0001** |
| Propachlor | 16 | 1.7 | 52 | 1.6 | 0.71 |
| Trifluralin | 32 | 3.4 | 75 | 2.2 | **0.04** |
| **Individual OP insecticides** |  |  |  |  |  |
| Chlorpyrifos | 10 | 1.1 | 17 | 0.5 | 0.05 |
| Diazinon | 17 | 1.8 | 86 | 2.6 | 0.20 |
| Dimethoate | 13 | 1.4 | 70 | 2.1 | 0.18 |
| Famphur | 8 | 0.9 | 31 | 0.9 | 0.86 |
| Fonofos | 9 | 1.0 | 41 | 1.2 | 0.53 |
| Malathion | 55 | 5.9 | 179 | 5.3 | 0.49 |
| Phorate | 13 | 1.4 | 45 | 1.3 | 0.89 |
| Terbufos | 10 | 1.1 | 39 | 1.2 | 0.83 |
| **Individual OC insecticides** |  |  |  |  |  |
| Aldrin | 2 | 0.2 | 66 | 2.0 | **0.0002** |
| Chlordane | 31 | 3.3 | 146 | 4.4 | 0.17 |
| Dieldrin | 2 | 0.2 | 54 | 1.6 | **0.0009** |
| DDT | 14 | 1.5 | 223 | 6.6 | **<0.0001** |
| Lindane | 9 | 1.0 | 82 | 2.4 | **0.006** |
| Methoxychlor | 82 | 8.8 | 169 | 5.0 | **<0.0001** |
| **Individual carbamate insecticides** |  |  |  |  |  |
| Carbaryl | 24 | 2.6 | 70 | 2.1 | 0.36 |
| Carbofuran | 29 | 3.1 | 78 | 2.3 | 0.17 |
| **Doctor diagnosed mononucleosis** |  |  |  |  |  |
| Yes | 88 | 9.5 | 37 | 1.1 | **<0.0001** |
| **Family history of lymphatic/haematopoietic cancer** |  |  |  |  |  |
| Yes | 27 | 2.9 | 147 | 4.4 | 0.10 |
| **Worked or lived on farmland** |  |  |  |  |  |
| Yes | 406 | 43.7 | 2132 | 63.5 | **<0.0001** |

**Notes:** ^a^ Based on Wald chi-square statistic; bold numbers indicate a statistically significant difference in proportions based on a significance level alpha=0.05

**Table S7.** Odds ratios (OR) and 95% confidence intervals (CI) for the association between number of pesticides used by functional and chemical group and Hodgkin lymphoma with proxy respondents excluded in the North American Pooled Project.

| **Pesticides** | **Cases** | **Controls** | **OR ^a^** | **95% CI** | |
| --- | --- | --- | --- | --- | --- |
| 0 | 280 | 1647 | 1.00 |  |  |
| 1 | 32 | 266 | 0.84 | 0.55 | 1.27 |
| 2 - 4 | 73 | 383 | 1.15 | 0.85 | 1.55 |
| 5+ | 37 | 245 | 1.03 | 0.69 | 1.54 |
| p-trend |  |  | 0.60 |  |  |
| **Fungicides** |  |  |  |  |  |
| 0 | 406 | 2475 | 1.00 |  |  |
| 1 | 11 | 55 | 0.89 | 0.44 | 1.81 |
| 2+ | 5 | 11 | 1.84 | 0.59 | 5.75 |
| p-trend |  |  | 0.58 |  |  |
| **Herbicides** |  |  |  |  |  |
| 0 | 304 | 1895 | 1.00 |  |  |
| 1 | 62 | 346 | 1.09 | 0.79 | 1.49 |
| 2 - 4 | 39 | 212 | 1.04 | 0.70 | 1.55 |
| 5+ | 17 | 88 | 1.32 | 0.74 | 2.37 |
| p-trend |  |  | 0.40 |  |  |
| **Insecticides** |  |  |  |  |  |
| 0 | 295 | 1749 | 1.00 |  |  |
| 1 | 62 | 420 | 1.01 | 0.73 | 1.39 |
| 2 - 4 | 49 | 288 | 1.07 | 0.75 | 1.52 |
| 5+ | 16 | 84 | **1.93** | **1.05** | **3.55** |
| p-trend |  |  | 0.18 |  |  |
| **Organophosphate insecticides** |  |  |  |  |  |
| 0 | 371 | 2281 | 1.00 |  |  |
| 1 | 31 | 155 | 1.10 | 0.71 | 1.69 |
| 2+ | 20 | 105 | 1.62 | 0.94 | 2.77 |
| p-trend |  |  | 0.10 |  |  |
| **Organochlorine insecticides** |  |  |  |  |  |
| 0 | 355 | 2155 | 1.00 |  |  |
| 1 | 42 | 217 | 1.10 | 0.76 | 1.61 |
| 2+ | 25 | 169 | 1.12 | 0.70 | 1.80 |
| p-trend |  |  | 0.53 |  |  |
| **Carbamate insecticides** |  |  |  |  |  |
| 0 | 298 | 2419 | 1.00 |  |  |
| 1 | 17 | 103 | 1.09 | 0.62 | 1.91 |
| 2+ | 7 | 19 | **2.61** | **1.00** | **6.86** |
| p-trend |  |  | 0.12 |  |  |

**Notes:** ^a^ adjusted for age group, sex, province or state of residence; bold text indicates a statistically significant results at alpha=0.05

**Table S8.** Odds ratios (OR) and 95% confidence intervals (CI) for the association between ever use of selected insecticides and Hodgkin lymphoma with proxy respondents excluded in the North American Pooled Project.

| **Pesticide** | **Cases** | **Controls** | **OR ^a^** | **95% CI** | |
| --- | --- | --- | --- | --- | --- |
| **Organophosphate** |  |  |  |  |  |
| Chlorpyrifos | 6 | 18 | 1.95 | 0.72 | 5.32 |
| Diazinon | 13 | 63 | 1.73 | 0.89 | 3.37 |
| Dimethoate | 13 | 57 | 1.56 | 0.80 | 3.04 |
| Famphur | 4 | 24 | 1.67 | 0.53 | 5.27 |
| Fonofos | 6 | 30 | 2.14 | 0.80 | 5.71 |
| Malathion | 34 | 170 | 1.21 | 0.79 | 1.85 |
| Phorate | 7 | 33 | 2.00 | 0.80 | 4.96 |
| Terbufos | 6 | 30 | 2.21 | 0.83 | 5.87 |
| **Organochlorine** |  |  |  |  |  |
| Aldrin | 5 | 47 | 1.63 | 0.61 | 4.38 |
| Chlordane | 19 | 131 | 0.88 | 0.52 | 1.50 |
| Dieldrin | 4 | 35 | 1.79 | 0.59 | 5.46 |
| DDT | 18 | 162 | 1.18 | 0.69 | 2.02 |
| Lindane | 10 | 60 | 1.68 | 0.80 | 3.54 |
| Methoxychlor | 42 | 191 | 0.99 | 0.67 | 1.45 |
| **Carbamate** |  |  |  |  |  |
| Carbaryl | 15 | 61 | 1.78 | 0.95 | 3.32 |
| Carbofuran | 13 | 70 | 1.34 | 0.70 | 2.56 |

**Notes**: ^a^ adjusted for age group, sex and location;

**Table S9.** Odds ratios (OR) and 95% confidence intervals (CI) for the association between ever use of selected herbicides and fungicides and Hodgkin lymphoma with proxy respondents excluded in the North American Pooled Project.

| **Pesticide** | **Cases** | **Controls** | **OR** | **95% CI** | |
| --- | --- | --- | --- | --- | --- |
| **Phenoxy herbicide** |  |  |  |  |  |
| 2,4-D | 80 | 432 | 1.18 | 0.88 | 1.58 |
| 2,4,5-T | 6 | 54 | 0.96 | 0.39 | 2.33 |
| MCPA | 12 | 54 | 1.12 | 0.56 | 2.23 |
| **Carbamate herbicide** |  |  |  |  |  |
| Diallate | 7 | 25 | 1.03 | 0.40 | 2.63 |
| Triallate | 6 | 15 | 1.95 | 0.67 | 5.69 |
| **Other herbicide** |  |  |  |  |  |
| Alachlor | 9 | 72 | 1.09 | 0.51 | 2.33 |
| Atrazine | 13 | 103 | 1.01 | 0.54 | 1.91 |
| Bromoxynil | 11 | 42 | 1.06 | 0.51 | 2.21 |
| Dicamba | 15 | 68 | 1.29 | 0.70 | 2.38 |
| Glyphosate | 39 | 165 | 1.02 | 0.68 | 1.53 |
| Propachlor | 6 | 44 | 1.22 | 0.49 | 3.06 |
| Trifluralin | 17 | 66 | 1.45 | 0.79 | 2.67 |
| **Fungicide** |  |  |  |  |  |
| Captan | 6 | 19 | 1.42 | 0.52 | 3.84 |
| Thiram | 9 | 36 | 0.94 | 0.42 | 2.09 |

**Notes**: ^a^ adjusted for age group, sex and location;

**Table S10.** Odds ratios (OR) and 95% confidence intervals (CI) for the association between number of pesticides used by functional and chemical group and Hodgkin lymphoma in mixed logistic regression models that included a random effects parameter for province or state of residence in the North American Pooled Project.

|  | *OR ^a^* | *95%CI* | |
| --- | --- | --- | --- |
| **Pesticides** |  |  |  |
| 0 | 1.00 |  |  |
| 1 | 0.86 | 0.52 | 1.42 |
| 2 - 4 | 1.07 | 0.69 | 1.65 |
| 5+ | 0.66 | 1.42 | 1.42 |
| **Fungicides** |  |  |  |
| 0 | 1.00 |  |  |
| 1 | 0.86 | 0.43 | 1.73 |
| 2+ | 1.85 | 0.60 | 5.73 |
| **Herbicides** |  |  |  |
| 0 | 1.00 |  |  |
| 1 | 0.82 | 0.45 | 1.49 |
| 2 - 4 | 0.79 | 0.42 | 1.50 |
| 5+ | 1.23 | 0.71 | 2.13 |
| **Insecticides** |  |  |  |
| 0 | 1.00 |  |  |
| 1 | 0.53 | 0.29 | 0.97 |
| 2 - 4 | 0.57 | 0.30 | 1.06 |
| 5+ | **1.76** | **1.01** | **3.07** |
| **Organophosphate insecticides** | | |  |
| 0 | 1.00 |  |  |
| 1 | 1.05 | 0.69 | 1.60 |
| 2+ | 1.55 | 0.94 | 2.55 |
| **Organochlorine insecticides** | | |  |
| 0 | 1.00 |  |  |
| 1 | 0.99 | 0.69 | 1.43 |
| 2+ | 1.09 | 0.70 | 1.71 |
| **Carbamate insecticides** | |  |  |
| 0 | 1.00 |  |  |
| 1 | 0.99 | 0.58 | 1.72 |
| 2+ | **2.40** | **1.01** | **5.73** |

**Notes**: ^a^ adjusted for age group, sex and respondent status; bold numbers indicate a statistically significant result at alpha=0.05

**Table S11.** Odds ratios (OR) and 95% confidence intervals (CI) for the association between ever use of selected insecticides and Hodgkin lymphoma in mixed logistic regression models that included a random effects parameter for province or state of residence in the North American Pooled Project.

| Pesticide | *OR ^a^* | *95%CI* | |
| --- | --- | --- | --- |
| **Organophosphate** |  |  |  |
| Chlorpyrifos | 1.73 | 0.61 | 4.95 |
| Diazinon | 1.66 | 0.89 | 3.09 |
| Dimethoate | 1.34 | 0.68 | 2.65 |
| Famphur | 2.16 | 0.84 | 5.60 |
| Fonofos | 1.81 | 0.71 | 4.63 |
| Malathion | 1.12 | 0.73 | 1.72 |
| Phorate | 1.55 | 0.65 | 3.70 |
| Terbufos | 2.25 | 0.92 | 5.46 |
| **Organochlorine** |  |  |  |
| Aldrin | 1.70 | 0.70 | 4.16 |
| Chlordane | 0.71 | 0.40 | 1.26 |
| Dieldrin | 1.99 | 0.75 | 5.30 |
| DDT | 1.23 | 0.75 | 2.03 |
| Lindane | 1.46 | 0.69 | 3.08 |
| Methoxychlor | 0.96 | 0.64 | 1.43 |
| **Carbamate** |  |  |  |
| Carbaryl | 1.70 | 0.91 | 3.18 |
| Carbofuran | 1.16 | 0.60 | 2.23 |

**Notes**: ^a^ adjusted for age group, sex and respondent status; bold numbers indicate a statistically significant result at alpha=0.05

**Table S12.** Odds ratios (OR) and 95% confidence intervals (CI) for the association between ever use of selected herbicides and fungicides and Hodgkin lymphoma in mixed logistic regression models that included a random effects parameter for province or state of residence in the North American Pooled Project.

| Pesticide | *OR ^a^* | *95%CI* | |
| --- | --- | --- | --- |
| **Phenoxy herbicide** |  |  |  |
| 2,4-D | 1.04 | 0.77 | 1.39 |
| 2,4,5-T | 0.82 | 0.34 | 1.97 |
| MCPA | 0.72 | 0.31 | 1.66 |
| **Carbamate herbicide** |  |  |  |
| Diallate | 1.27 | 0.50 | 3.23 |
| Triallate | 1.95 | 0.63 | 6.08 |
| **Other herbicide** |  |  |  |
| Alachlor | 1.14 | 0.58 | 2.27 |
| Atrazine | 0.94 | 0.52 | 1.72 |
| Bromoxynil | 0.84 | 0.37 | 1.87 |
| Dicamba | 1.06 | 0.55 | 2.02 |
| Glyphosate | 0.93 | 0.61 | 1.42 |
| Propachlor | 1.17 | 0.51 | 2.73 |
| Trifluralin | 1.12 | 0.59 | 2.15 |
| **Fungicide** |  |  |  |
| Thiram | 1.07 | 0.47 | 2.47 |

**Notes**: ^a^ adjusted for age group, sex and respondent status; bold numbers indicate a statistically significant result at alpha=0.05

**Table S13.** Odds ratio (OR) and 95% confidence intervals (CI) for the association between duration (years of use) and frequency (days/year) of use of select pesticides and Hodgkin lymphoma in mixed logistic regression models that included a random effects parameter for province or state of residence in the North American Pooled Project.

| **Pesticide** | OR | 95% CI | |
| --- | --- | --- | --- |
| **Organophosphate insecticide** |  |  |  |
| **Malathion** |  |  |  |
| *Duration of use* |  |  |  |
| 0 | 1.00 |  |  |
| 1-5 | **1.74** | **1.02** | **2.95** |
| ≥ 6 years | 0.94 | 0.46 | 1.89 |
| p-trend | 0.08 |  |  |
|  |  |  |  |
| *Frequency of use* |  |  |  |
| 0 | 1.00 |  |  |
| 1-2 | 1.33 | 0.76 | 2.32 |
| ≥ 3 days / year | 0.94 | 0.41 | 2.16 |
| p-trend | 0.14 |  |  |
|  |  |  |  |
| **Organochlorine insecticide** |  |  |  |
| **Methoxychlor** |  |  |  |
| *Duration of use* |  |  |  |
| 0 | 1.00 |  |  |
| 1-5 | 0.88 | 0.43 | 1.80 |
| ≥ 6 years | 0.93 | 0.58 | 1.49 |
| p-trend | **0.04** |  |  |
|  |  |  |  |
| *Frequency of use* |  |  |  |
| 0 | 1.00 |  |  |
| 1-2 | 0.53 | 0.25 | 1.15 |
| ≥ 3 days / year | 1.22 | 0.76 | 1.96 |
| p-trend | 0.11 |  |  |
|  |  |  |  |
| **Phenoxy herbicide** |  |  |  |
| **2,4-D** |  |  |  |
| *Duration of use* |  |  |  |
| 0 | 1.00 |  |  |
| 1-5 | 0.93 | 0.59 | 1.48 |
| ≥ 6 years | 1.18 | 0.80 | 1.74 |
| p-trend | 0.05 |  |  |
|  |  |  |  |
| *Frequency of use* |  |  |  |
| 0 | 1.00 |  |  |
| 1-2 | 0.83 | 0.53 | 1.29 |
| ≥ 3 days / year | 1.18 | 0.76 | 1.83 |
| p-trend | 0.10 |  |  |
|  |  |  |  |
| **Organophosphate herbicide** |  |  |  |
| **Glyphosate** |  |  |  |
| *Duration of use* |  |  |  |
| 0 | 1.00 |  |  |
| 1-5 | 0.96 | 0.58 | 1.58 |
| ≥ 6 years | 1.41 | 0.76 | 2.64 |
| p-trend | 0.08 |  |  |
|  |  |  |  |
| *Frequency of use* |  |  |  |
| 0 | 1.00 |  |  |
| 1-2 | 1.05 | 0.65 | 1.72 |
| ≥ 3 days / year | 1.56 | 0.81 | 3.02 |
| p-trend | 0.18 |  |  |

**REFERENCES**

1. Textor J, van der Zander B, Gilthorpe MS, Liskiewicz M, Ellison GT. Robust causal inference using directed acyclic graphs: the R package 'dagitty'. *Int J Epidemiol.* 2016;45(6):1887-1894.
